# Dataset of the use of tannin of néré (parkia-biglobosa) as a solution for the sustainability of the soil constructions in West Africa

**DOI:** 10.1016/j.dib.2016.05.072

**Published:** 2016-06-04

**Authors:** Sinko Banakinao, Sonnou Tiem, Komlan Lolo, Yao Koutsawa, Kofi-Sa Bedja

**Affiliations:** a Laboratoire génie-civil, ENSI, Université de Lomé, Togo; b Laboratoire génie-électrique, ENSI, Université de Lomé, Togo; c Laboratoire génie-mécanique, ENSI, Université de Lomé, Togo; d Materials Research and Technology Department, Luxembourg Institute of Science and Technology, 41, rue du Brill, L-4422 Belvaux, Luxembourg

**Keywords:** Parkia-biglobosa, Bipinnateleaves, Mimosaceae

## Abstract

Soil is the main material of construction in African rural areas. Sustainability of construction with soil is a thorny problem to any builder. Finding ways to improve the durability of soil is not only essential but also salutary for the African rural community that mostly lives in soil-built houses that are very often short-lived. The present data assessed the resistance to simple compression and the resistance to bad weather by simulating rainfall alternations through a test of cycles of alternate watering and drying of blocks built from four types of different soils without binder, along with blocks built from the same soils.

**Specifications Table**

**Table:** 

Subject area	Construction and building material
More specific subject area	Physical and mechanical characteristics of the soils
Types of data	Chart, figure
How data were acquired	Simple compression tests (standard NFP18-411), Alternate wetting and drying.
Data format	Analyzed
Experimental factors	Four types of soil have been collected from stacked heaps in four towns in Togo; the husk of the néré is collected from farmers and grinded into powder.
Experimental features	Simple compression tests and durability experiments by alternate watering and drying.
Data source location	**Mango**, (GPS coordinates 31P X=204969 Y=1168103), **Blitta** (GPS coordinates 31P X=279562 Y=926241), **Sotouboua** (GPS coordinates 31P X=278406Y=936764) and **Lomé** (GPS coordinates 31P X=345051 Y=702831), Togo, West Africa
Data accessibility	Data are within this article

**Value of the data**

• These data are very important for the improvement of the mechanical and waterproof characteristics (resistance to simple compression) of soils by the introduction of an eco-friendly and economic vegetable binder.
• They can open the way to new soil stabilization techniques using plant-based binders hydrolyzable tannins.
• These data can improve traditional techniques earth building and may be a solution for constructing more sustainable soil, as well as contributing in a remarkable way to the restoration and renovation of historic soil monuments.

## 1 Data

Data presented here by charts and figures, describes the experiment of simple compression and the experiment of durability by humidification and drying on non-stabilized and stabilized blocks built from four types of different soils. The néré pod is used for stabilization.

## 2 Experimental design, materials and methods

### 2.1 Sites selected

The experiment was conducted on four (4) different soil samples collected in Togo in West Africa, located between the latitudes 6°06 and 11°08 North and the longitudes 0°09 West and 1°49 East. The immediate neighboring countries are the Republic of Benin in the East, the Republic of Ghana in the West, and the Republic of Burkina Faso in the North. In the South, it is bordered by the Atlantic Ocean in the Gulf of Benin, which is, itself, in the Gulf of Guinea on the West African coast. Soil samples have been taken from four cities in Togo: **Mango**, (GPS coordinates 31P *X*=204969 *Y*=1168103), **Blitta** (GPS coordinates 31P *X*=279562 *Y*=926241), **Sotouboua** (GPS coordinates 31P *X*=278406 *Y*=936764) and **Lomé** (GPS coordinates 31P *X*=345051 *Y*=702831).The husk of the nére that is used as a binder has been found in one of these cities, Sotouboua.

### 2.2 Sample collection and process of searching for the husk of the néré

**Fig. 1:**
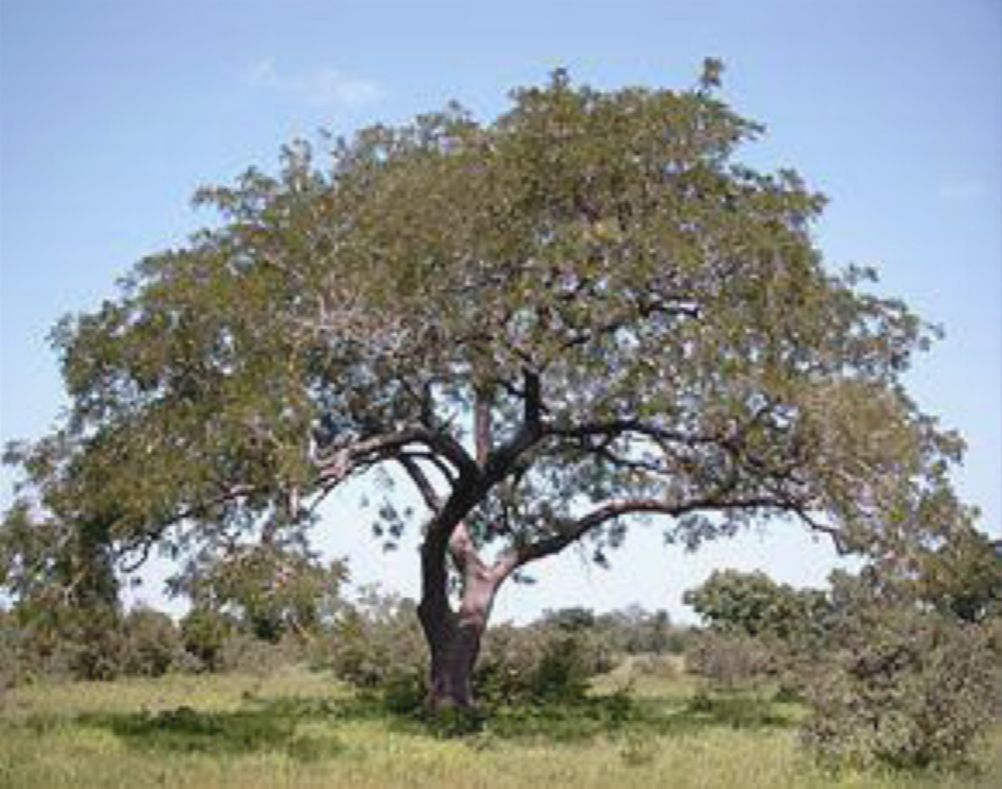
Photo of the néré tree.

**Fig. 2:**
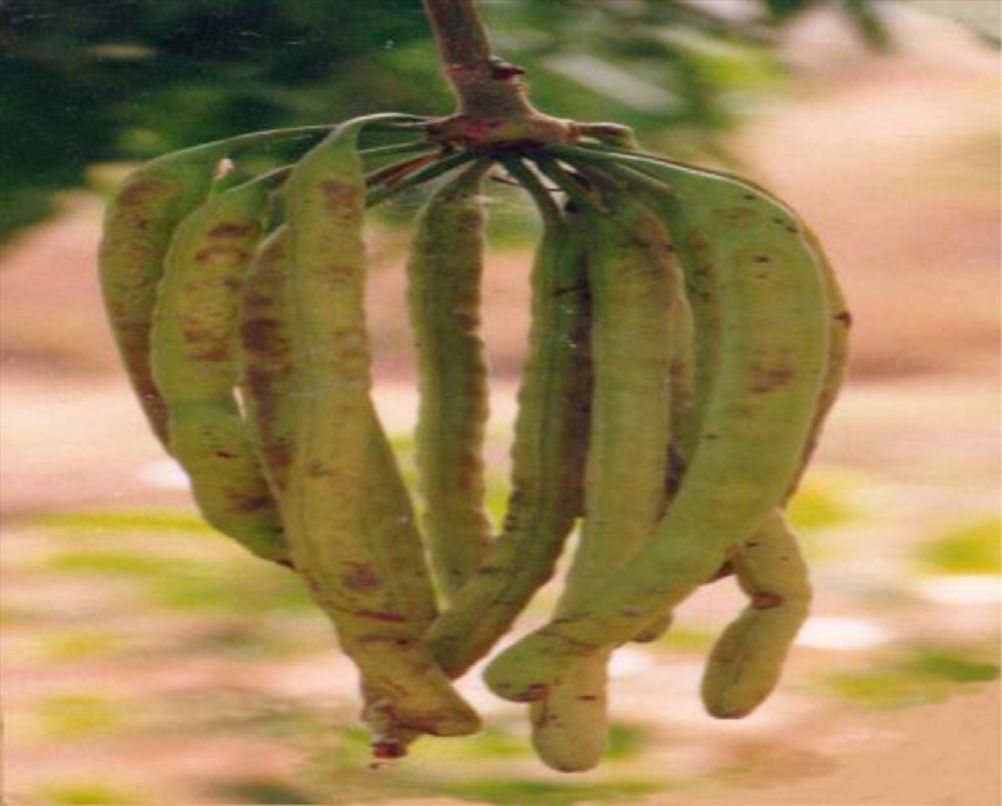
Photo of the florescence of the néré.

The husk of the néré comes from the néré tree known scientifically as Pakia biglobosa. (Fig. 1, Fig. 2).

**Fig. 3:**
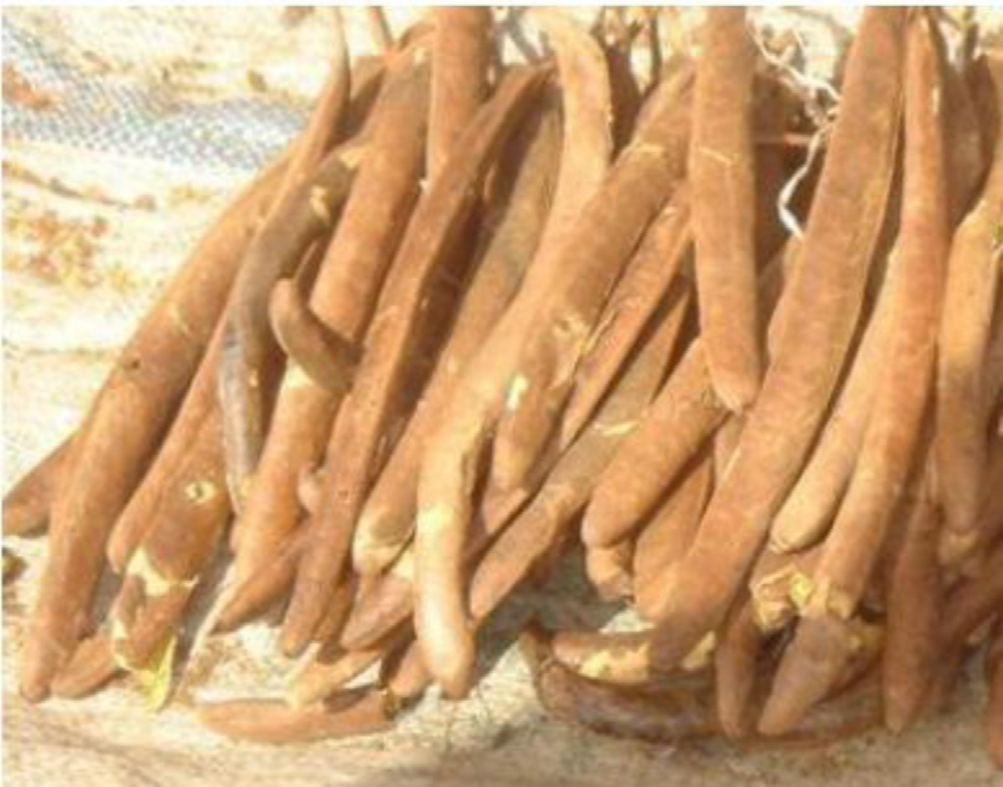
Photo of the cloves of the néré.

**Fig. 4:**
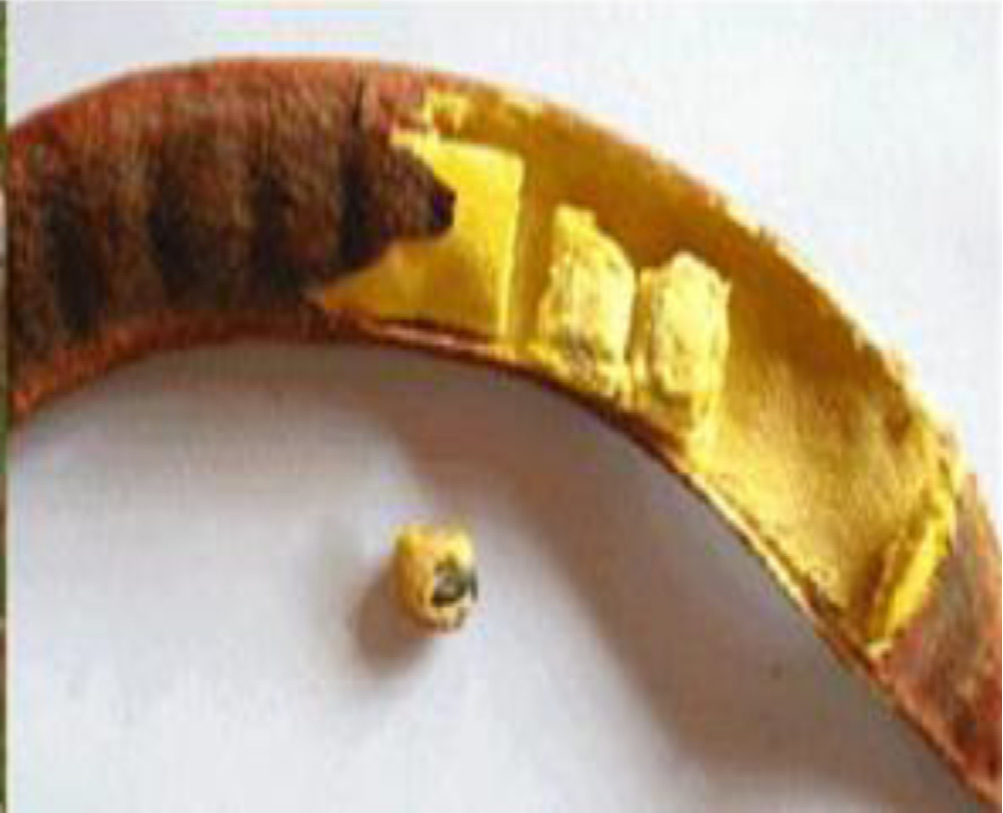
Photo of the pulp of the néré.

**Fig. 5:**
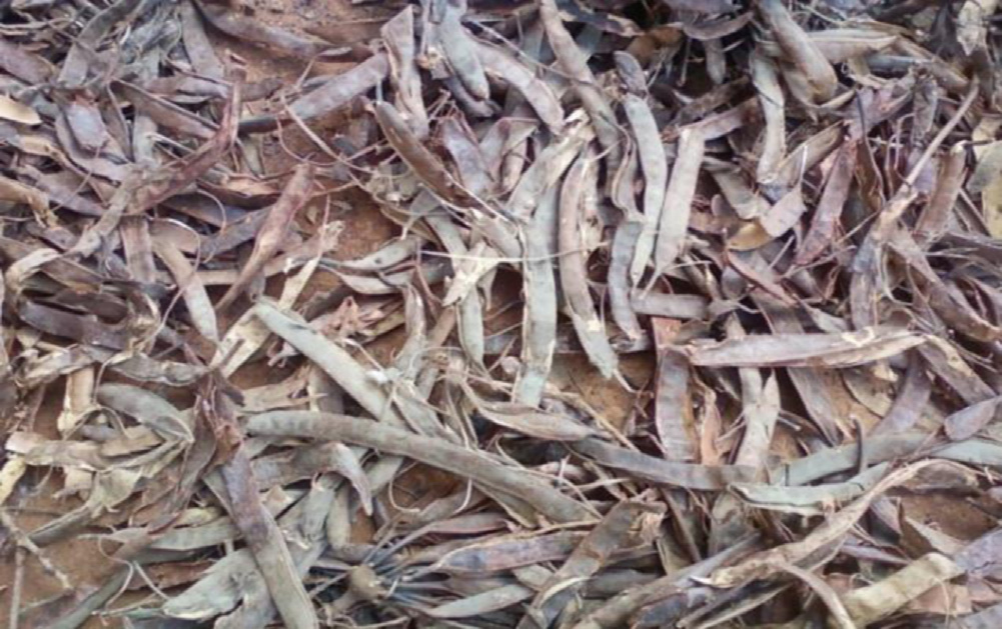
Photo of the husk of the néré.

**Fig. 6:**
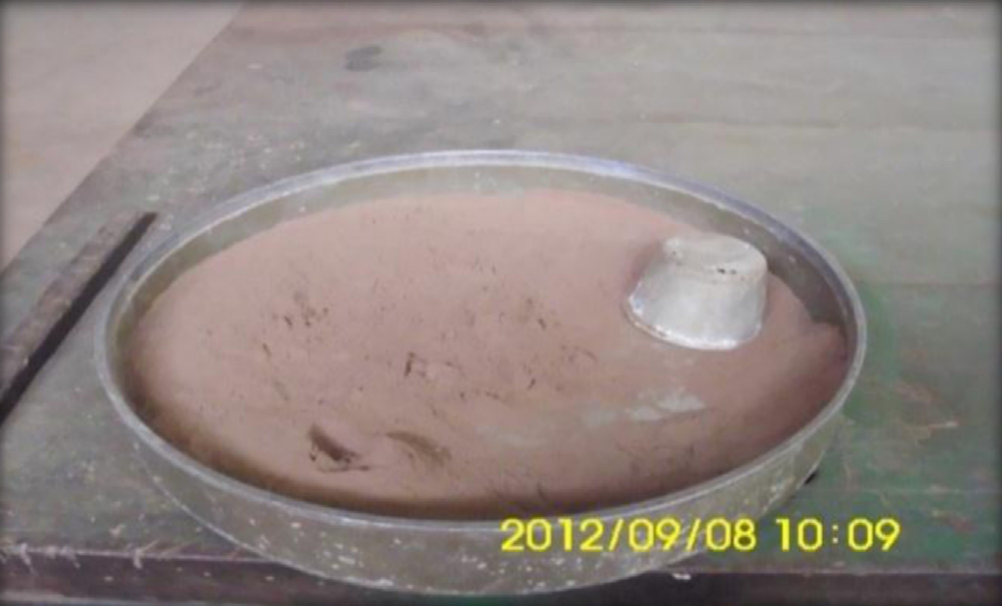
Photo of the powder of the néré.

Once the clove of the néré (Fig. 3) is harvested, the yellow pulp (Fig. 4) is extracted for juice, and the seed is used to make traditional mustard known as dawa-dawa, tchoti, etc. The residue of the husk of the néré (Fig. 5) is ready to be used as waste or burnt. Therefore, this material is collected for the production of the husk powder of the néré. The husk of the néré is heated to 150 °C for three (3) hours. It is cut into pieces, crushed in a RETSCH-type knife crusher, and passed through 2-mm and 0.125-mm sieves. The powder retrieved from the process (Fig. 6) is used as a binder to be mixed with the soil or used to make tannic extraction.

### 2.3 Construction of the composite soil-pod material of the néré

The composite soil-pod material of the néré is gained from a mixture of soil and néré pod in appropriate proportions. When we increase the quantity of the husk powder of the néré in the composite soil-pod material and process it according to the methodology of the Proctor test and CBR experiment, we notice that the load-bearing capacity rises to a maximum and drops, which enables us to determine the optimal rate of the husk of the néré that offers the maximal load-bearing capacity, this rate is used to construct blocks from the soil-pod of the néré.

### 2.4 Construction of blocks from the soil-pod of the néré

A mass of soil mixed with an optimal quantity of the husk of the néré is homogenized and humidified with an optimal quantity of water. This composite material is conserved in the shade for at least 24 h. Afterwards, the material is compressed in a press at a pressure of 3.5 MPa. We gain cubic blocks of 10 cm long, 10 cm large and 10 cm thick. These blocks are dried and underwent an experiment of simple compression with a press to determine the CBR with values ranging from approximately 0.0024 kN to 50 kN. For our data, 32 blocks from each soil were constructed, which consisted of 16 blocks for each bare soil without the néré husk and 16 blocks from each soil mixed with an optimal percentage of the néré husk. After drying the blocks in the sun for 7 days, we tested eight blocks from each soil and determined their water content. Therefore, we noticed that the stabilized blocks have a resistance higher than non-stabilized blocks, but the content in water is not zero. After steaming at 105 °C for 24 h, we tested eight stabilized blocks and eight non-stabilized blocks and measured their water content, which is almost nil.

**Chart 1:**
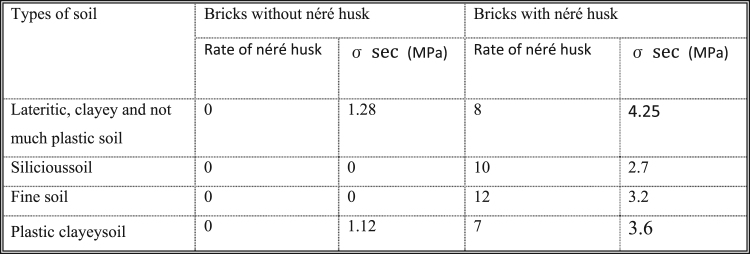
Experimental data of mechanical resistance of compressed stabilized and non-stabilized blocks.

The observed data are included in Chart 1.

We observed that the simple compressive strength of the stabilized bricks is higher than that of non-stabilized bricks. [1,2] Thus, néré husk contains tannins that add to the simple compressive strength of bricks. Tannins [3,4] behave like a binder in soil, increasing the cohesion between the soil seeds and therefore increasing the mechanical resistance.

### 2.5 Experiment of durability by humidification and drying or cycle of alternate watering and drying

The type of the durability experiment (watering–drying) is a simulation of rainfall alternations (water saturation in winter, drying in summer). This experiment is performed for the purpose of studying the durability and the resistance when the material is humid and is improved by the néré husk, as well as when the material is bare without the néré husk when they are subject to seasonal rainfall alternations. We applied it to the four types of soil. For each soil, 32 cubic blocks have been constructed, of which a series of 16 blocks do not contain the néré husk, while another series of 16 blocks were stabilized with the néré husk. All 32 blocks of each type of soil were dried in the sun for 7 days, and they underwent five cycles of alternate watering and drying.

## 3 First cycle of drying and watering

**Fig. 7:**
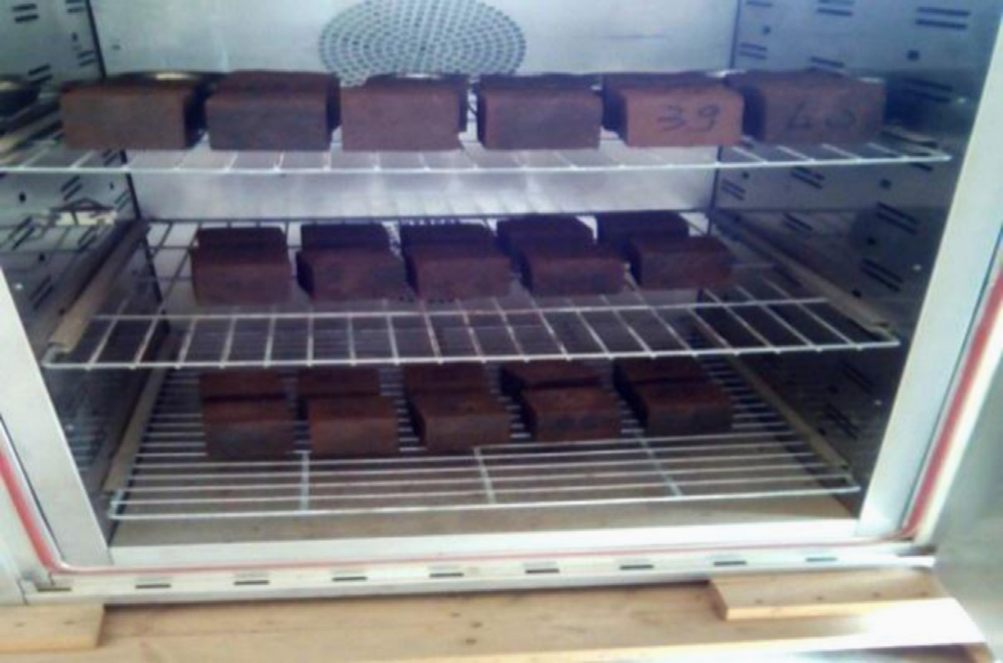
Block in an oven.

**Fig. 8:**
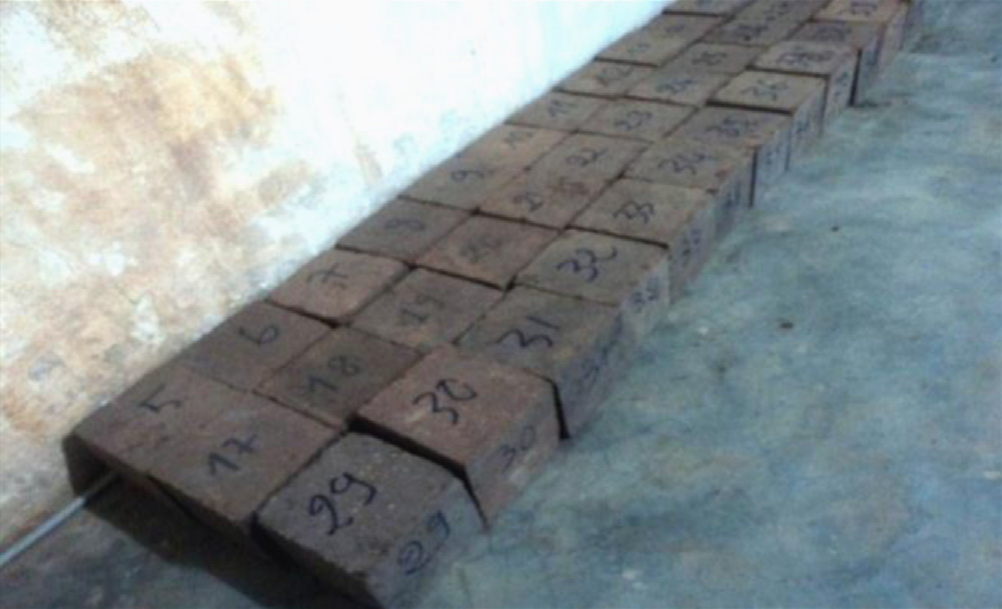
Block out of an oven.

**Drying**: Thirty-two blocks of each type of the soil were placed in an oven at 105 °C for 24 h (Fig. 7). Out of the oven (Fig. 8), eight non-stabilized blocks and eight stabilized blocks of each type of the soil were grinded. We notice that the resistance to the compression of the stabilized blocks was higher than the resistance to the compression of the blocks of non-stabilized blocks.

**Fig. 9:**
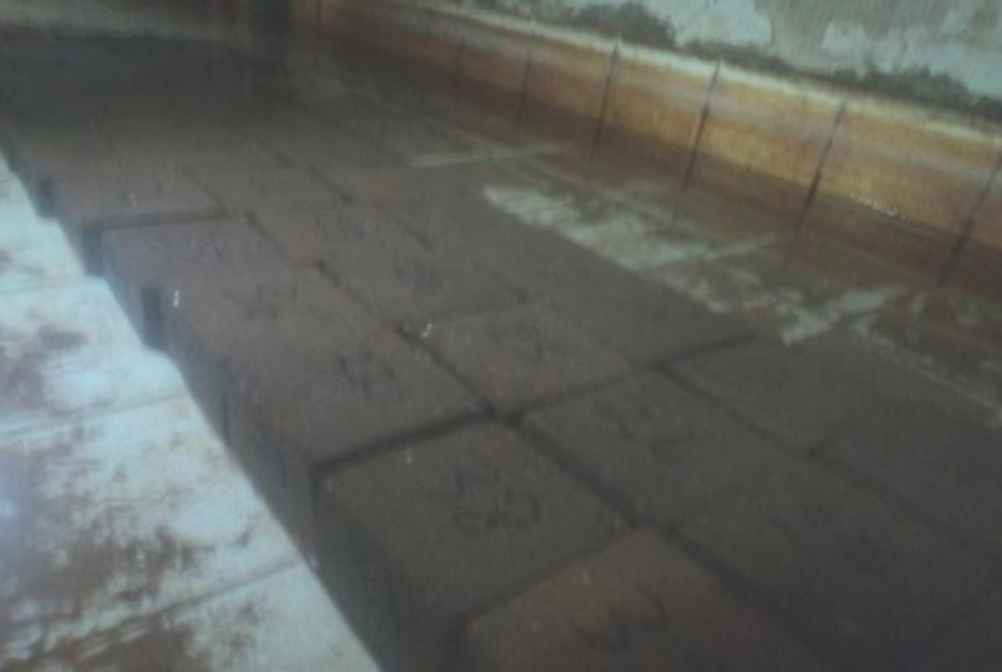
Immersion of blocks.

**Fig. 10:**
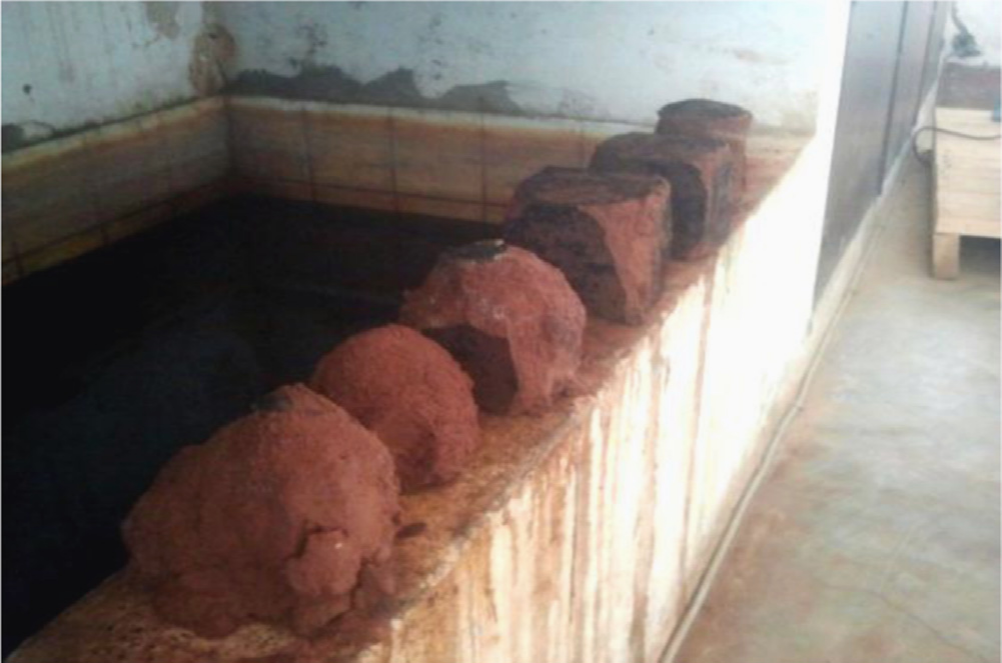
Non-stabilized blocks after 1st cycle of immersion.

**Fig. 11:**
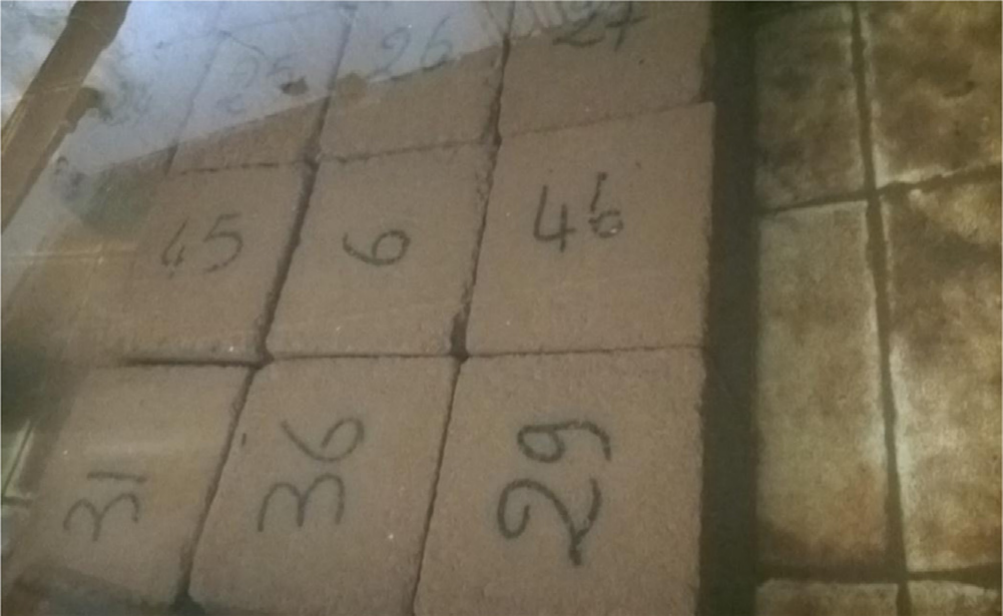
Stabilized blocks after the 1st cycle of immersion.

**Watering**: The remaining 16 blocks, eight non-stabilized blocks and eight stabilized blocks of each type of soil, were immersed in water whose temperature is 26 °C for five hours (Fig. 9). The duration of immersion must be higher than the time of full saturation of blocks. After five hours of watering, we observed the following:

• The non-stabilized blocks of all of the soils, in part or in whole, dissolved in water (Fig. 10).
• However, all of the stabilized blocks remained intact (Fig. 11).

## 4 Second, third, fourth and fifth cycle of drying and watering

**Fig. 12:**
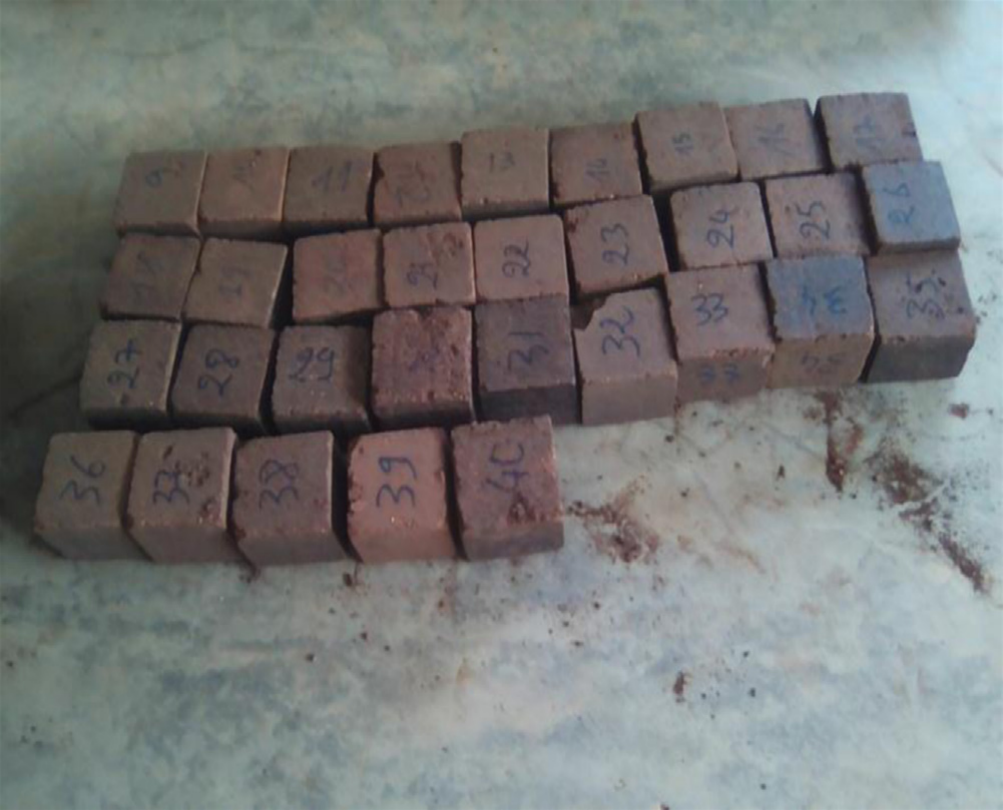
Stabilized blocks after fifth cycle of drying and watering.

For the second cycle, only eight stabilized blocks of each type of soil were left for experimental purposes. They go successively to the third, fourth and fifth cycle of watering and drying. We noticed that the edges of the blocks of silty and clay soil crumbled, while the blocks of lateritic soils did not suffer any damage (Fig. 12). All of the blocks were crushed and removed from the water after the fifth cycle. We noticed that the resistance to a simple compression after the five cycles is higher than the resistance to simple compression of dried blocks before immersion.

## 5 Determining of coefficient of resistance to humidity (*C*_rh_)

The coefficient of resistance to humidity (*C*_rh_) is defined with respect to the resistance to crushing after five cycles of alternate watering and drying and the resistance to the simple compression when dried.

(1) $$C_{rh}=\frac{\sigma \;\mathrm{msa}}{\sigma \;\sec}$$

C_rh_: Coefficient of resistance to humidity,

σ msa**:** The resistance to humid compression (resistance after immersion at the fifth cycle).

(2) $$\sigma \;\mathrm{msa}=\frac{F\;\mathrm{msa}}{S}$$

σ sec**:** The resistance to the compression at drying (resistance after steaming at the first cycle).

(3) $$\sigma \;\sec=\frac{F\;\sec}{S}$$

F sec: The maximal charge of breaking-up after steaming at the first cycle.

F msa: The maximal charge of breaking-up after immersion at the fifth cycle.

**Chart 2:**
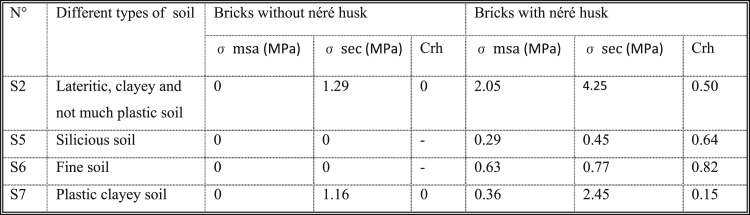
Data from test of resistance to humidity.

The observed data are included in the following below Chart 2.

The chart provides the following information:

• The resistance to wet compression (σmsa) is nil for all of the non-stabilized soils (Chart 2), but it is not nil when the same soils are stabilized at the néré husk.
• The resistance to dry compression (σsec) is nil for all of the powdered soils (fine soil and silty soil), but it is different from zero for coherent soils (Lateritic, clayey and not much plastic soil). The coefficient of resistance to humidity (Crh) is nil when the soils are non-stabilized and different from zero when the soils are stabilized with néré husk.
• The stabilized soils are more waterproof than the non-stabilized soils.
